# Enhanced green fluorescent protein-mediated synthesis of biocompatible graphene

**DOI:** 10.1186/s12951-014-0041-9

**Published:** 2014-10-03

**Authors:** Sangiliyandi Gurunathan, Jae Woong Han, Eunsu Kim, Deug-Nam Kwon, Jin-Ki Park, Jin-Hoi Kim

**Affiliations:** Department of Animal Biotechnology, Konkuk University, 1 Hwayang-Dong, Gwangin-gu Seoul, 143-701 South Korea; GS Institute of Bio and Nanotechnology, Coimbatore, Tamil Nadu 641024 India; Animal Biotechnology Division, National Institute of Animal Science, Suwon, 441-350 Korea

**Keywords:** Enhanced green fluorescent protein, Graphene oxide, Graphene, Human embryonic kidney 293 cells, Cell viability, Membrane leakage, Oxidative stress

## Abstract

**Background:**

Graphene is the 2D form of carbon that exists as a single layer of atoms arranged in a honeycomb lattice and has attracted great interest in the last decade in view of its physical, chemical, electrical, elastic, thermal, and biocompatible properties. The objective of this study was to synthesize an environmentally friendly and simple methodology for the preparation of graphene using a recombinant enhanced green fluorescent protein (EGFP).

**Results:**

The successful reduction of GO to graphene was confirmed using UV–vis spectroscopy, and FT-IR. DLS and SEM were employed to demonstrate the particle size and surface morphology of GO and EGFP-rGO. The results from Raman spectroscopy suggest the removal of oxygen-containing functional groups from the surface of GO and formation of graphene with defects. The biocompatibility analysis of GO and EGFP-rGO in human embryonic kidney (HEK) 293 cells suggests that GO induces significant concentration-dependent cell toxicity in HEK cells, whereas graphene exerts no adverse effects on HEK cells even at a higher concentration (100 μg/mL).

**Conclusions:**

Altogether, our findings suggest that recombinant EGFP can be used as a reducing and stabilizing agent for the preparation of biocompatible graphene. The novelty and originality of this work is that it describes a safe, simple, and environmentally friendly method for the production of graphene using recombinant enhanced green fluorescent protein. Furthermore, the synthesized graphene shows excellent biocompatibility with HEK cells; therefore, biologically synthesized graphene can be used for biomedical applications. To the best of our knowledge, this is the first and novel report describing the synthesis of graphene using recombinant EGFP.

## Background

Graphene has a two-dimensional (2-D) nanostructure with a single layer of carbon atoms and has attracted much interest in recent years because of its unique mechanical, thermal, catalytic, electronic, optical, and biological properties [1-4]. Graphene and graphene-based materials have been widely used in several applications including bio-sensing [5], antibacterial compositions [6-8], drug delivery [9], tissue scaffolds [10], catalysis [11], and energy storage [12]. The production of graphene in large quantities using an environmentally friendly approach is essential but also a significant challenge [13].

Several methods have been established for the synthesis of graphene and its derivatives, including exfoliation of graphite (Gt) [14], flash reduction [15], hydrothermal dehydration [16], mechanical exfoliation [3], epitaxial growth [17], photocatalysis [18], and photodegradation [19]. Although several methods are available for the preparation of graphene, solution-based chemical reduction of graphene oxide (GO) to graphene is considered one of the most efficient methods for low-cost and large-scale production of graphene [13]. Reduction of GO by chemical methods seems to be promising, because of the low cost and potential for large-scale production. Such methods are also appropriate for chemical modification and subsequent processing. However, in chemical methods, the use of hydrazine and hydrazine derivatives as strong reducing agents for the formation of graphene can be toxic or explosive, resulting in challenges for large-scale production. The resulting graphene also possesses very limited solubility or even irreversible agglomeration during preparation in water and most organic solvents unless capping reagents are used owing to the strong π–π stacking tendency between rGO sheets [20,21]. To overcome the aggregation and solubility problems, several polymers or surfactants have been used, such as poly(N-vinyl-2-pyrrolidone) [22], poly(sodium-4-styrene sulfonate) [23], poly(allylamine) [24], and potassium hydroxide [25]. Recently, Akhavan et al. [26] demonstrated a possible route for inexpensive mass production of high-quality graphene sheets from natural and industrial carbonaceous wastes.

The toxicity of GO and graphene has been studied in various cell types such as neuronal cells [27], lung epithelial cells [28], fibroblasts [29], primary mouse embryonic fibroblast cells [30], and cancer cells [31], and the results vary across cell and material types.

Surface modification of graphene has been reported to alter its toxicity [31], with reduced GO and carboxylated graphene reported to be less toxic than GO or native graphene [32]. Single-layer GO sheets were found to be internalized and sequestered in cytoplasmic, membrane-bound vacuoles in human lung epithelial cells and fibroblasts, with toxicity induced at concentrations above 20 μg/mL after 24 h [27,29]. Sanchez et al. [4] reported that graphene-family nanomaterials (GFNs) can be either benign or toxic to cells, and that the biological responses depend on layer number, lateral size, stiffness, hydrophobicity, surface functionalization, and concentration. In addition, the biocompatibility and cytotoxicity depend on the type of reducing agent used for the functionalization of GO.

Graphene has been used as a possible biocompatible nanocarrier for delivering drugs [33] and also as a functional biomaterial. Sun et al. [9] reported that non-toxic PEGylated nano-graphene oxide could deliver water-insoluble cancer drugs. Fan et al. [34] showed that graphene/chitosan composites were biocompatible in L929 cells and that the absence of metallic impurities in graphene sheets makes them potential candidates as scaffolds for tissue engineering. Furthermore, Chen et al. [35] reported that graphene oxide (GO)/ultra-high-molecular-weight polyethylene (GO/UHMWPE) composites showed remarkably enhanced hardness and slightly improved yield strength compared with pure UHMWPE. The addition of small amounts of GO did not affect the attachment and proliferation of MC3T3-E1 osteoblasts cultured on GO/UHMWPE composite surfaces, indicating its excellent biocompatibility. Akhavan et al. [36] reported size-dependent cyto- and genotoxic effects of reduced graphene oxide nanoplatelets (rGONPs) rGONPs on cells. A cell viability test showed significant cell death on treatment with 1.0 μg/mL rGONPs with an average lateral dimension (ALD) of 11 ± 4 nm, whereas rGO sheets an ALD of 3.8 ± 0.4 μm exhibited a significant cytotoxic effect only at the high concentration of 100 μg/mL after 1 h of exposure time. Akhavan et al. [37] demonstrated the size-dependent cytotoxic and genotoxic effects of reduced graphene oxide nanoplatelets on human mesenchymal stem cells (hMSCs). Furthermore, Akhavan et al. [38] used ginseng extract-reduced GO to differentiate stem cells. Park et al. [39] used graphene-as a substrate to promote human neural stem cell adhesion and differentiation into neurons. Lee et al. [40] reported that the strong non-covalent binding ability of graphene allows it to act as a pre-concentration platform for osteogenic inducers, which accelerate the differentiation of mesenchymal stem cells (MSCs) growing on it toward the osteogenic lineage. Akhavan et al. [37] used graphene nanogrids as two-dimensional selective templates for accelerated differentiation of human MSCs (hMSCs) isolated from umbilical cord blood into osteogenic lineages. The biocompatible and hydrophilic graphene nanogrids showed high actin cytoskeleton expression coinciding with the patterns of the nanogrids. Akhavan and Ghaderi [41] introduced a reduced graphene oxide (rGO)/TiO_2_ heterojunction film as a biocompatible flash photo stimulator for the effective differentiation of hNSCs into neurons. Graphene nanogrids on a SiO_2_ matrix containing TiO_2_ nanoparticles (NPs) were also applied as a photocatalytic stimulator to accelerate the differentiation of human neural stem cells (hNSCs) into two-dimensional neural networks [42].

Several environmentally friendly methods have been developed using various biomolecules such as ascorbic acid [43], amino acids [44], glucose [45], and bovine serum albumin [46] as reducing agents or stabilizers. In addition, microorganisms have also used to reduce GO, including *Shewanella* [47], *Escherichia coli* [48,49], *Pseudomonas aeruginosa* [8], *Bacillus marisflavi* [50], and *Ganoderma* spp [21]. Some purified proteins have also been used for synthesis of graphene, such as melatonin [51], l-glutathione [52], and humanin [53]. Recently, the synthesis of graphene has been increased significantly because of the wide range of resources and availability of simple, cost-effective, and environmentally friendly approaches. The major problem encountered during the synthesis of nanoparticles using biomass is the isolation and purification of the nanoparticles from the biomass, which requires many downstream processing steps including sonication and ultracentrifugation to attain maximum yield [54]. Moreover, endotoxin may be present in the nanoparticles, which may limit the use of the nanoparticles in medical applications [55]. Therefore, this study attempted to use a recombinant protein.

Recombinant enhanced green fluorescent protein (EGFP) (Gene Bank Accession no. U57607) is a protein composed of 293 amino acid residues (32.7 kDa) that has an isoelectric point of 6.2 and exhibits bright green fluorescence when exposed to light in the blue to ultraviolet range. EGFP has been widely used as a biological reporter to identify tissue and cells with target gene expression [56,57]. Previous studies showed no obvious detrimental effects of EGFP and no toxicity, i.e., it is biologically inert [58,59]. In addition, EGFP was selected here as a reducing and stabilizing agent for synthesis of graphene because it is a natural protein from the jellyfish *Aequorea victoria* and has been proven to be an excellent biological reporter [60]. Thus, without any other toxic reagents added, the raw material and reaction products are all environmentally friendly, which should increase the efficiency and large-scale synthesis of graphene. Additionally, EGFP contains five cysteine amino acid residues, each containing a thiol group that can be oxidized to form the disulfide derivative cysteine, which functions as a nucleophile [61]. Protons have high binding affinity to oxygen-containing groups, such as hydroxyl and epoxide groups on GO, resulting in the formation of H_2_O molecules [27,62]. The unique chemical structure of EGFP makes it not only an ideal reducing agent but also an effective capping agent. Therefore, we addressed the following objectives: first, the development of a simple, dependable, and environmentally friendly approach for synthesis of graphene using recombinant EGFP; second, the characterization of GO and EGFP-reduced GO; and finally, the evaluation of cellular responses of GO and EGFP-rGO in human embryonic kidney 293 cells.

## Results and discussion

### Synthesis and characterization of EGFP-rGO

**Figure 1:**
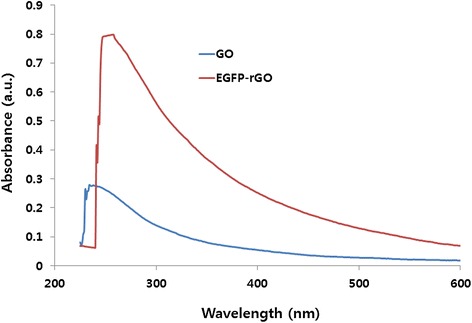
**Synthesis and characterization of GO and EGFP-rGO by ultraviolet–visible spectroscopy.** Spectra of GO exhibited a maximum absorption peak at approximately 230 nm, which corresponds to a π-π transition of aromatic C–C bonds. The absorption peak for reduced GO was red-shifted to 258 nm. At least three independent experiments were performed for each sample and reproducible results were obtained. Data from a representative experiment are shown.

As shown in Figure 1, EGFP-rGO was synthesized by a two-step process, including an oxidation step and an EGFP-based reduction step. In the first step, graphene oxide was formed by the oxidation of graphite crystals according to a modification of the Hummers method [63]; the crystals were dispersible in water. In the second step, a stable black aqueous suspension was obtained through a chemical deoxidization process by using EGFP as both a reducer and a stabilizer. Similarly, Wang et al. [13] reported a simple method of reduction of GO to rGO using the natural polymer heparin as both a reducing agent and a stabilizer to produce a stable aqueous suspension of heparin-rGO sheets. Fan et al. [34] fabricated biocompatible graphene-reinforced chitosan composites in which chitosan was significantly reinforced by the addition of a small amount of graphene sheets. The graphene/chitosan composites were biocompatible in the L929 fibrosarcoma cell line.

The reduction of GO was confirmed using UV–vis absorption spectroscopy. As shown in Figure 1, the absorption peak of the GO dispersion was located at 230 nm with a shoulder peak at about 300 nm, which was consistent with previous reports [13,27,62]. After the reduction process, the peak was red-shifted to 258 nm and the absorbance was increased dramatically in the entire spectral region. This result suggests that GO was reduced by EGFP and that the aromatic structure of graphene may be restored. Further evidence showed that the UV–vis absorption spectrum of GO was characterized by the *π*–*π*∗ of the C = C plasmon peak at approximately 230 nm and a shoulder at approximately 300 nm that is often attributed to *n*–*π*∗ transitions of the carbonyl groups [62,64]. With reduction by EGFP, the plasmon peak gradually red-shifted to approximately 258 nm, indicating the restoration of sp^2^ carbon and possible rearrangement of atoms [65]. Similar trends were also observed for the reduction of GO by L-ascorbic acid [43,66], L-cysteine [62], melatonin [51], heparin [13], dopamine [67], and humanin [53].

### FTIR spectra of GO and EGFP-rGO

**Figure 2:**
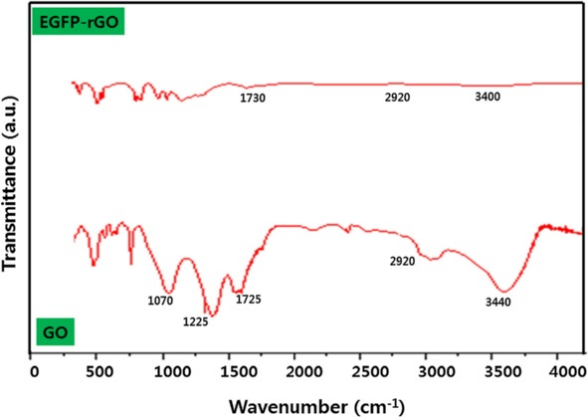
**Fourier transform infrared spectroscopy spectra of GO and EGFP-rGO.**

The reduction of oxygen-containing functional groups of GO by EGFP was confirmed by FT-IR spectroscopy. Figure 2 shows the FT-IR spectra of GO and EGFP-rGO. The presence of different types of oxygen-containing groups in graphene oxide was confirmed at 3440 cm^−1^ (O-H stretching vibrations), 1725 cm^−1^ (stretching vibrations from C = O), 1225 cm^−1^ (C-OH stretching vibrations), and 1070 cm^−1^ (C-O stretching vibrations), as reported earlier [68,69]. In addition, the substitution of hydroxyl groups on the GO surface by carboxyl groups was confirmed by the CH_2_-stretching vibration at 2,920 cm^−1^ (lower spectrum) [70]. In contrast, the FT-IR spectrum of graphene completely differs from that of GO. The FTIR peak of EGFP-rGO showed O-H stretching vibrations, stretching vibrations from C = O, C-OH stretching vibrations, and C-O stretching vibrations at 3440, 1725, 1225, and 1070 cm^−1^, respectively, indicating that GO was significantly reduced by the deoxygenation procedure. The intensities of absorption peaks corresponding to oxygen functional groups decreased and these functional groups almost disappeared. Altogether, these results clearly confirm that the oxygen-containing groups were removed during reduction using EGFP. These changes in EGFP-rGO compared with GO in FT-IR spectra were identical with those of earlier reports that used various reducing agents such as hydrazine [14], vitamin C [66], L-cysteine [62], heparin [13], and humanin [53].

### XRD analysis of GO and EGFP-rGO

**Figure 3:**
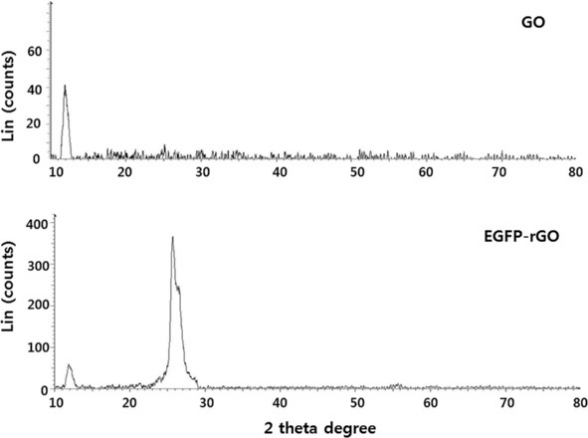
**XRD patterns of GO and EGFP-rGO.** In the XRD pattern of GO (top panel), the strong and sharp peak at 2θ = 11.7° corresponds to an interlayer distance of 7.6 Å. EGFP-rGO (bottom panel) has a broad peak centered at 2θ = 25.8°, which corresponds to an interlayer distance of 3.6 Å. These XRD results are related to the reduction of GO by EGFP and the process of removing intercalated water molecules and oxide groups. At least three independent experiments were performed for each sample and reproducible results were obtained. Data from a representative experiment are shown.

To further characterize the crystal structures, the XRD patterns of the exfoliated GO and EGFP-rGO were studied. The characteristic peak of GO appears at 11.7°, corresponding to a d-spacing of 0.76 nm resulting from the formation of hydroxyl, epoxy, and carboxyl groups (Figure 3). In contrast to GO, EGFP-rGO showed no peaks at 11.7°, which indicates that most of the oxygen functional groups of GO were removed. Compared with pristine graphite (2θ = 26.4°), the diffraction peak of exfoliated GO moved to 11.7° (002) with a layer-to-layer distance (d-spacing) of 0.76 nm. This value was larger than the d-spacing of pristine graphite (0.34 nm) because of the introduction of numerous oxygenated functional groups on the carbon sheets [13]. After the exfoliated GO was reduced by EGFP, the peak at 11.7° disappeared, but a new diffraction peak appeared at 2θ = 25.8° with a d-spacing of 0.36 nm, which was closer to the typical (002) diffraction peak of graphite (2θ = 26.4°, d-spacing of 0.34 nm). The higher interlayer spacing value of exfoliated GO resulted from the introduction of numerous oxygenated functional groups on the carbon sheets [7,21,48]. The data obtained from this experiment suggest that EGFP played an important role in the deoxygenation of GO and also that the reduction of GO by EGFP was consistent with earlier reports using various reducing agents including vitamin C [66], L-cysteine [62], heparin [13], and humanin [53].

### Size distribution analysis of GO and EGFP-rGO

**Figure 4:**
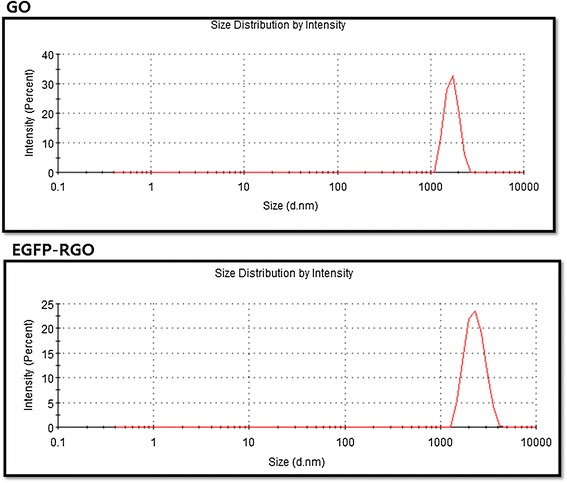
**Size distribution analysis of GO and EGFP-rGO.** Aqueous dispersions of GO and EGFP-rGO were characterized by DLS analysis using a particle size analyzer at the scattering angle θ = 90°. The data show the average values from triplicate measurements. The sample concentrations were all 250 μg/mL.

Size distribution analysis was performed to elucidate the state of GO and EGFP-rGO in an aqueous solution using DLS measurement [71] with a concentration of 250 μg/mL. The average hydrodynamic diameter (AHD) of GO and EGFP-rGO was 2288 ± 20 nm and 2607 ± 32 nm, respectively (Figure 4). However, after the reduction of GO with EGFP, the AHD increased and was relatively larger than that of GO. This obvious change of size distribution suggests that EGFP not only acted as a reducing agent to prepare rGO but also functionalized on the surface of the resulting rGO. Similar results were observed for heparin and biopolymer-functionalized reduced graphene oxide [13,72]. Graphene nanoplates functionalized with isocyanate showed the effective hydrodynamic diameter size of 560 ± 60 nm. Lammel et al. [73] reported that the hydrodynamic diameter of GO functionalized with carboxyl graphene nanoplatelets increased from 385 to 1,110 nm. Liu et al. [74] reported that aqueously dispersed graphite (Gt), graphite oxide (GtO), graphene oxide (GO), and reduced graphene oxide (rGO) had sizes of 5,250, 4,420, 560, and 2,930 nm, respectively. A similar trend was observed for GO reduced by *Pseudomonas aeruginosa* [8], *Bacillus marisflavi* [50]*, Ginkgo biloba* [70], and *Ganoderma* spp [21]. The size of EGFP-rGO was slightly larger than that of GO, indicating that EGFP not only acted as a reducing agent but also was functionalized on the surfaces of the resulting rGO, leading to an increased size [75]. Similarly, Wang et al. [13] found that the average size of heparin-reduced graphene oxide was larger than that of GO under the same experimental conditions. Altogether, our data and data from other groups suggest that EGFP used as a reducing agent plays an important role in increasing the size of rGO.

### Surface properties of GO and EGFP-rGO

**Figure 5:**
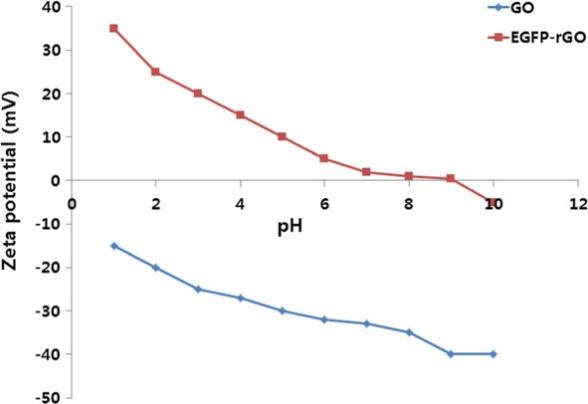
**Zeta potential of as-prepared GO and EGFP-rGO as a function of pH, in aqueous dispersions at a concentration of ~0.05 mg ml** ^**−1**^ **.**

Zeta potential is an important factor for characterizing the dispersion stability of colloids because the magnitude and sign of the effective surface charge associated with the double layer around the colloid, and it directly influences the electrostatic interaction between different graphene sheets [76,77]. Zeta potential measurements were carried out in aqueous solutions of the GO and EGFP-rGO in function of pH is important to determine the surface charge of the sheets (Figure 5). The results show that GO sheets are highly negative charged with an average −29.7 mV at pH range between 2 and 10. This value is attributed to the presence of oxygen species at the surface of GO. On the contrary, EGFP-rGO, shows positive zeta potential values for the same pH range, which is suggest that the lower charge density of this type of graphene. Interestingly, recombinant proteins treated GO sheets resulted in the reduction and almost complete elimination of the oxygen functionalities at the surface of graphene materials.

### Surface morphology analysis of GO and EGFP-rGO by SEM

**Figure 6:**
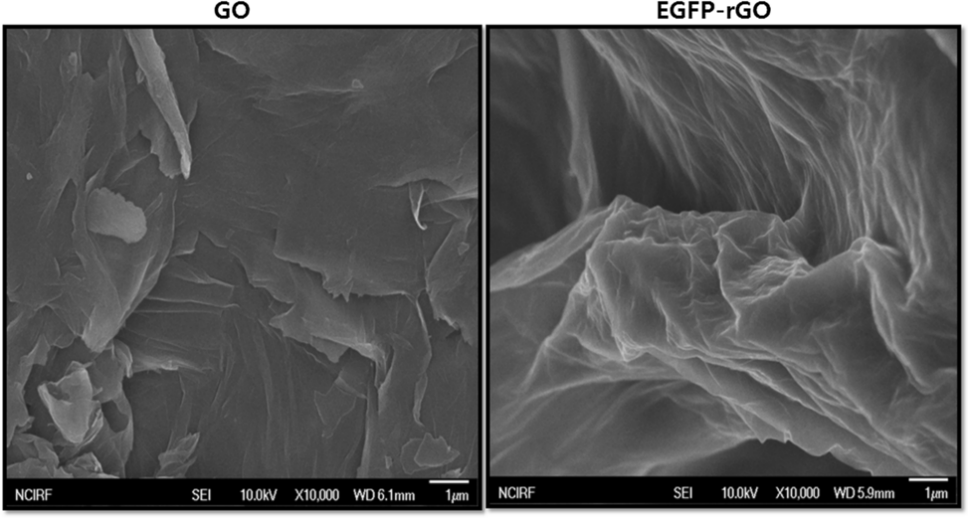
**SEM images of GO and EGFP-rGO.** Representative SEM images of GO and EGFP-rGO dispersions at 500 μg/mL.

The surface morphology of the GO and EGFP-rGO samples was analyzed using SEM. As shown in Figure 6A, the GO samples contain several layers of sheets, and further the sheets are aggregated and crumpled sheets are closely associated with each other to form a continuous conducting network. The edges of the GO sheets appeared crumpled, folded, and closely restacked with one another because of the oxidation process [78]. Jeong et al. [79] reported that at higher concentrations, the surfaces of GO sheets have a soft-carpet-like morphology, possibly because of the presence of residual H_2_O molecules and hydroxyl or carboxyl groups attached to GO. In contrast to GO, on SEM the EGFP-rGO samples resemble transparent and rippled silk waves (Figure 6B). He and Gao [80] reported that Gt appears to pile up in thick cakes, whereas GO is exfoliated into thin large flakes with wavy wrinkles. Previously, we observed on SEM that GO consisted of individual sheets closely associated with each other, with a silky and leaf-like structure, whereas *Ginkgo biloba* extract-reduced GO (Gb-rGO) sheets showed thin layers of nanosheets and were mainly comprised of larger, wavy forms [70]. The graphene sheets were found to possess a curled morphology consisting of a thin, wrinkled, paper-like structure, with fewer layers (approximately four layers) and a large specific surface area [81]. Graphene nanosheets were functionalized with long chains and polymers, resulting in coarse and hairy surfaces with blurry edges of the flakes [80]. Previously, we reported using SEM that GO was present as multilayered, wavy, folded flakes, whereas fungal extract-reduced graphene oxide showed several layers stacked on top of one another similarly to sheets of paper, with a silky, wrinkled, and flower-like curling morphology [70]. This difference in morphology between the folded, stacked structure of GO and transparent and rippled silk wave structure of graphene suggests that EGFP played an important role in the reduction of GO to graphene. The data obtained from this study suggest that synthesis of graphene using biological molecules was similar to that of graphene sheets prepared from Gt powder through oxidation followed by rapid thermal expansion in a nitrogen atmosphere [81].

### Raman spectroscopy analysis of GO and EGFP-rGO

**Figure 7:**
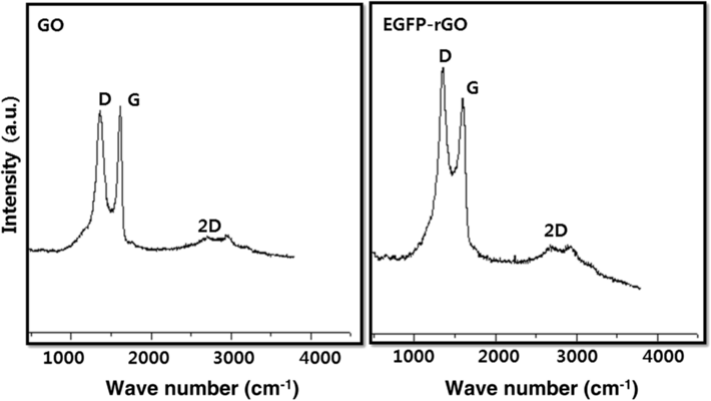
**Raman spectroscopy analyses of GO and EGFP-rGO samples.** Raman spectra were obtained using a laser excitation of 532 nm at a power of, 1 mW. The figure shows representative Raman spectra of GO and EGFP-rGO samples after removal of the fluorescent background. The intensity ratios of the D-peak to the G-peak were 1.8 and 2.149 for GO and EGFP-rGO, respectively. At least three independent experiments were performed for each sample and reproducible results were obtained.

Raman spectroscopy is used to characterize the structural electronic properties of graphite and graphene-based materials [21,82,83]. Raman spectra are also used to measure induced enormous structural changes during chemical oxidation of pristine graphite and the reduction of GO to rGO [83]. In the Raman spectra, the G band resulting from first-order scattering of the E_2g_ phonons of sp^2^ carbon atoms and the D band originating from the breathing mode of k-point photons of A_1g_ symmetry are the two main characteristic features of graphene-based materials [84-86]. In the Raman spectrum of GO, the G band is broadened and shifted to 1615 cm^−1^. In addition, the D band at 1359 cm^−1^ becomes prominent, indicating a reduction in the size of the in-plane sp^2^ domains, possibly because of extensive oxidation-induced defects in the sheets (Figure 7). The Raman spectrum of the rGO reduced by EGFP also contains both G and D bands located at 1607 and 1351 cm^−1^, respectively; however, the D/G intensity ratio (2.149) is increased compared to that in GO upon reduction. This change suggests a decrease in the average size of the sp^2^ domains upon reduction of the exfoliated GO [14,84].

The major effects of deoxygenation are the restoration of the sp^2^ network and the introduction of small and isolated aromatic domains, and these effects are responsible for the observed increase in the ID/IG ratio in rGO [66,83,86,87]. Wang et al. [82] suggested that the G band is broadened and shifted upward to 1,595 cm^−1^, and the increased intensity of the D band at 1,350 cm^−1^ could be attributed to the significant decrease in the size of the in-plane sp^2^ domains resulting from oxidation and ultrasonic exfoliation, in addition to the partially ordered graphite crystal structure of graphene nanosheets. The Raman spectra of graphene-based materials also show a two-dimensional (2D) band that is sensitive to the stacking of graphene sheets. It is well known that the two-phonon (2D) Raman scattering of graphene-based materials is useful to differentiate monolayer graphene from multilayer graphene as it is highly sensitive to the stacking of graphene layers [14,88,89]. Another characteristic of single-layer graphene is the relatively strong Raman intensity of the 2D band with respect to the G-band [90]. Usually, a Lorentzian peak for the 2D band of monolayer graphene sheets is observed at 2,679 cm^−1^, whereas this peak is broadened and shifted to a higher wave number in the case of multilayer graphene [14,88,89]. We observed the 2D band at 2699 cm^−1^, which is the same as the previously reported peak position for single-layer graphene [90,91]. Thus, our sample could consist of single-layer graphene flakes.

It should be noted that this ratio is higher than those reported for rGO produced using various reducing agents such as L-cysteine [62], dextran [92], baker’s yeast [93], DTT [83,94], and NaBH_4_ [95]. The Raman spectroscopy analyses described here agree with those of previous studies that used various biomolecules and organisms to reduce GO to graphene, such as L-cysteine [62], Baker’s yeast [93], heparin [13], *Escherichia coli* [48], *P. aeruginosa* [8], Humanin [53], *Ganoderma* spp [21], and *Ginkgo biloba* [70].

### Biocompatibility of GO and EGFP-rGO

**Figure 8:**
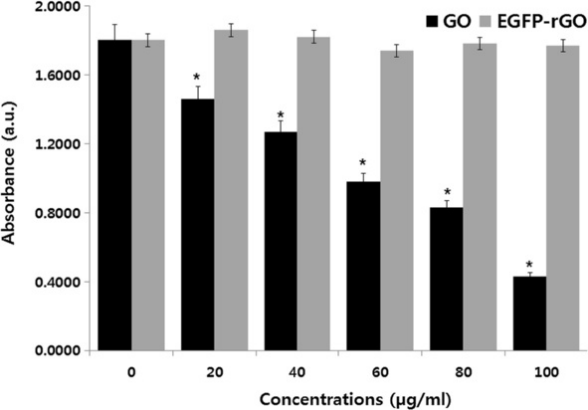
**Effects of GO and EGFP-rGO on cell viability of human embryonic kidney 293 cells.** Cell viability of human kidney cells was determined using WST-8 assay after 24 hours exposure to different concentrations of GO or EGFP-rGO. The results represent the means of three separate experiments, and error bars represent the standard error of the mean. GO- and EGFP-rGO-treated groups showed statistically significant differences from the control group by the Student’s *t*-test (*P* < 0.05).

The HEK cell line has been extensively used as an expression tool for recombinant proteins [96]. Therefore, we used the HEK cell line as a model system to study the effect of GO and EGFP-rGO. Figure 8 shows the biocompatibility of EGFP-rGO in HEK cells assessed using the WST assay. GO exhibited concentration-dependent toxicity compared to untreated control cells, whereas EGFP-rGO-treated cells showed no significant toxicity when compared to untreated cells. Several studies have shown interactions between dispersed graphene and GO sheets in various cell types such as monolayer cultures of neuronal cells [27], lung epithelial cells [28], fibroblasts [30,47], and human breast cancer cells [21]. Single-layer GO sheets were found to be internalized and sequestered in cytoplasmic, membrane-bound vacuoles by human lung epithelial cells or fibroblasts, and they induced toxicity at concentrations above 20 μg/mL after 24 h [28,29,94,97]. Limited literature is available on the biocompatibility of graphene [4]. GFNs have been suggested to be useful as biosensors [98], tissue scaffolds [10], carriers for drug delivery and gene therapy [99], antibacterial agents [7,8], and bio-imaging probes [27] because of their unique features over other types of nanomaterials, including their high specific surface area, which allows high-density biofunctionalization and drug loading. The results from our study indicate that EGFP-rGO can be used as a biocompatible material. Altogether, the results from our study and those from other groups suggest that EGFP-rGO can be used in various biomedical applications.

### Effect of EGFP-rGO on LDH leakage

**Figure 9:**
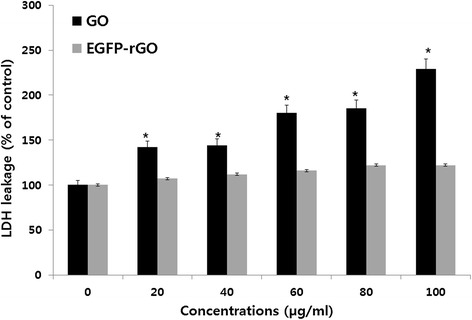
**Effects of GO and EGFP-rGO on lactate dehydrogenase activity in human embryonic kidney 293 cells.** Lactate dehydrogenase activity was measured at 490 nm, using the cytotoxicity detection lactate dehydrogenase kit. The results represent the means of three separate experiments, and error bars represent the standard error of the mean. GO- and EGFP-rGO-treated groups showed statistically significant differences from the control group by the Student’s *t*-test (*P* < 0.05).

LDH (lactate dehydrogenase) is present in all types of cells and LDH leakage is a useful index for cytotoxicity on the basis of loss of membrane integrity, a hallmark of necrosis [100]. Based on the percentage of the maximum LDH release, in the present study EGFP-rGO was considered non-toxic to cells, whereas GO showed toxicity to the cells in a concentration-dependent manner when compared to untreated cells (Figure 9). Significant LDH release was observed after 24 h of exposure to GO at higher concentrations, whereas graphene had no effect on the release of LDH. Thus, the LDH assay results were consistent with the cell-viability assay results. The toxicity of graphene materials depends on their size, shape, composition, surface charge, and surface chemistry, in addition to the target cell type [101]. Zhang et al. [27] observed that graphene aggregates/agglomerates that had sedimented onto the surface of rat PC12 cells caused an increase in LDH leakage only at the highest exposure concentration (100 μg/mL). Our earlier findings also suggest that at higher concentrations, TEA-rGO has no significant toxicity in mouse embryonic fibroblast cells [102]. Therefore, EGFP-derived graphene is also biocompatible.

### Effects of EGFP-rGO on oxidative stress

**Figure 10:**
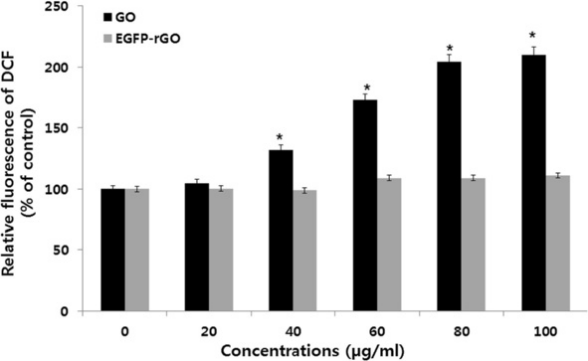
**Effects of GO and EGFP-rGO on generation of ROS in human embryonic kidney 293 cells.** The relative fluorescence of 2’,7’-dichlorofluorescein was measured using a spectrofluorometer with excitation at 485 nm and emission at 530 nm. The results represent the means of three separate experiments and the error bars represent the standard error of the mean. Treated groups GO, showed statistically significant differences from the control group, as determined by Student’s *t*-test (*P* < 0.05).

The DCF assay was performed to investigate the toxicity of nanomaterials attributable to ROS generation. Following exposure of HEK cells for 24 h to GO and EGFP-rGO, the state of oxidative stress in the cells was observed. As shown in Figure 10, the ROS generation increased in a concentration-dependent manner as the concentration of GO was increased, whereas EGFP-rGO had no significant impact, even at high concentrations, when treated cells were compared to untreated cells. These results were consistent with the results from the WST-8 assay and LDH assay, suggesting that toxicity in cells exposed to GO may result from oxidative stress mediated by ROS generation. It was previously shown that exposure to multiwalled carbon nanotubes (MWCNTs) resulted in a concentration-dependent cytotoxicity in cultured human embryonic kidney cells, which was associated with increased oxidative stress [103]. Zhang et al. [104] reported that surface functionalization (e.g., PEGylation) of single-walled carbon nanotubes (SWCNTs) reduced the ROS-mediated toxicological response in PC-12 cells. Induction of oxidative stress is considered to be one of the principal mechanisms underlying nanomaterial toxicity [105]. Lammel et al. [73] demonstrated that GO and carboxyl graphene nanoplatelets (CXYG) induce the generation of intracellular ROS in a concentration- and time-dependent manner in the human hepatocellular carcinoma cell line HepG2. GO-mediated cell death is caused by increased intracellular ROS levels originating from mitochondrial damage [73]. Stern et al. [106] suggest that several nanomaterials cause cell death through autophagy and lysosomal dysfunction. Qu et al. [107] reported that ROS production was independent of surface modification on QDs and that ROS did not account for the cytotoxicity of QD-PEG-NH_2_ particles in J774A.1 cells. Recently, Wu et al. [108] investigated the toxicity of graphene oxide in *Caenorhabditis elegans* at adult day 10 and found that prolonged exposure to 0.1 mg/L GO did not induce the noticeable intestinal autofluorescence or intestinal ROS production compared with the control; however, prolonged exposure to 10–100 mg/L GO resulted in intestinal autofluorescence and intestinal ROS production. Chong et al. [109] assessed the effect of graphene quantum dots (GQD) using various measures such as cell viability, cell apoptosis and necrosis, and LDH and ROS levels, and found that over 95% and 85% of HeLa cells and A549 cells, respectively, remained alive after 24 h of incubation with GQD-PEG, even when the GQD concentration increased to 160 μg/mL. Furthermore, they suggested that the low cytotoxicity resulted from PEGylation or the inherent properties of the GQD sample. Graphene nanoparticles, depending on the synthesis method, can exhibit different morphologies, chemical properties, and physical properties. Earlier studies also suggest that graphene nanoparticles show diverse responses in cells and tissues depending on their morphology and synthesis method [110].

### Effect of EGFP-rGO on cell morphology

**Figure 11:**
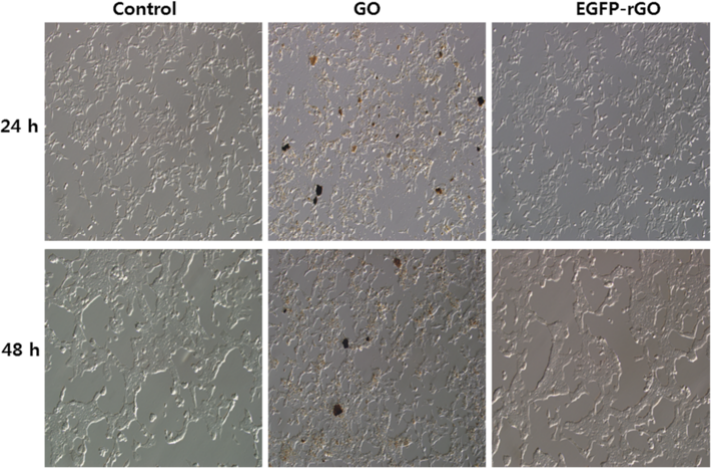
**The effect of GO and EGFP-rGO on morphology of human embryonic kidney 293 cells.** The microscopy images of human kidney cells were treated with concentrations of GO and EGFP-rGO (100 μg/ml) for 24 hours.

Biocompatibility is important for the development of new nanomaterials for biological and biomedical applications [50]. In addition to the biochemical assays described above, we evaluated the morphology of the cells treated with GO and EGFP-rGO. The effect of EGFP-rGO on cell morphology was determined using higher concentrations of GO and EGFP-rGO (100 μg/mL), and the cells were seeded at the same density of 1 × 10^4^ cells per plate. After 24 and 48 h of incubation, we observed the morphology of cells, and surprisingly, EGFP-rGO had no apparent effect; the cells were healthy (Figure 11); conversely, GO-treated cells were unhealthy, and the structure of the cells was contracted (Figure 11). Cheng et al. [67] reported that biopolymer-functionalized rGO exhibits an ultralow hemolysis ratio and significant cytocompatibility in human umbilical vein endothelial cells (HUVECs), even at a high concentration of 100 μg/mL. Talukdar et al. [71] evaluated the effect of various types of graphene materials such as graphene nano-onions (GNOs), graphene oxide nanoribbons (GONRs), and graphene oxide nanoplatelets (GONPs) on the viability and differentiation of human mesenchymal stem cells (MSCs). They found that the cytotoxic effect was concentration-dependent but not time-dependent. In our study, concentrations lower than 50 μg/mL showed no significant differences compared to untreated controls. Our data suggest that EGFP-rGO at up to 100 μg/mL has no effect on cell viability, LDH, ROS generation, or on cell morphology. Our earlier studies demonstrated both cytotoxicity and biocompatibility of graphene materials in various cell types. Altogether, our findings and those of other research groups suggest that the cytotoxicity or biocompatibility of graphene materials is dependent on physicochemical properties such as the density of functional groups, size, and conductivity, in addition to the type of reducing agents used for the deoxygenation of GO, degree of functionalization, and cell type [50,75]. Finally, graphene materials prepared using recombinant EGFP could be useful for potential biomedical applications.

## Conclusion

Commonly, the reduction of GO using chemical reducing agents is harmful to human health and the environment, and aggregation is another problem that occurs during the reduction process. Here, we show the synthesis of biocompatible graphene using recombinant EGFP. EGFP is one of the most widely used tools in biology because of its stability and lack of toxicity. In the present study, we explored the potential application of EGFP for a different purpose other than the tagging usually reported in the literature. We have developed a simple, dependable, and environmentally friendly method for the fabrication of reduced GO. Our findings suggest that GO induced significant concentration-dependent decreases in the viability of HEK cells, whereas graphene exerted no toxic effects on HEK cells at a concentration of 100 μg/mL. Therefore, it is concluded that the use of a biological substrate in a simple and environmentally friendly approach for synthesis of graphene resulted in significant deoxygenation of suspended GO suspensions, thus providing a suitable substitute for chemical reducing agents and potentially enabling biomedical applications of graphene-based materials. This work may provide additional insight into graphene synthesis.

## Materials and methods

### Materials

Gt powder, NaOH, KMnO4, NaNO_3_ anhydrous ethanol, 98% H_2_SO_4_, 36% HCl, and 30% H_2_O_2_ aqueous solution were purchased from Sigma-Aldrich (St Louis, MO, USA). Penicillin-streptomycin solution, trypsin-ethylenediaminetetraacetic acid solution, Dulbecco’s Modified Eagle Medium (DMEM), and 1% antibiotic-antimycotic solution were obtained from Gibco (Life Technologies, Carlsbad, CA, USA). Fetal bovine serum and the in vitro toxicology assay kit were purchased from Sigma-Aldrich. Enhanced green fluorescent protein was purchased from Bio-vision (Cat.No. 4999–100; Milpitas, California, USA).

### Synthesis of GO

GO was synthesized as described previously [21,57]. In a typical synthesis process, natural Gt powder (2 g) was added to cooled (0°C) H_2_SO_4_ (350 mL), and then KMnO_4_ (8 g) and NaNO_3_ (1 g) were added gradually while stirring. The mixture was transferred to a 40°C water bath and stirred for 60 min. Deionized water (250 mL) was slowly added and the temperature was increased to 98°C. The mixture was maintained at 98°C for a further 30 minutes and the reaction was terminated by the addition of deionized water (500 mL) and 30% H_2_O_2_ solution (40 mL). The color of the mixture changed to brilliant yellow, indicating the oxidation of pristine Gt to Gt oxide. The mixture was then filtered and washed with diluted HCl to remove metal ions. Finally, the product was washed repeatedly with distilled water until pH 7.0 was achieved, and the synthesized Gt oxide was further sonicated by ultrasonication for 30 min.

### Preparation of EGFP-rGO

Reduction of GO was performed as described previously [21,41] with suitable modifications. Using GO as a precursor, EGFP-rGO was prepared using EGFP as both a reducing agent and a stabilizer. In a typical procedure, reduced GO (rGO) was obtained from the reaction of EGFP with GO. A mixed aqueous solution containing EGFP (100 μg/mL) and GO (1 mg/mL) was ultrasonicated for 15 min, and the mixture was maintained at 40°C for 1 h. The mixture was then cooled to room temperature and ultrasonicated for a further 15 min. After being vigorously stirred for 5 min, the mixture was stirred in a water bath (90°C) for 1 h. The resulting stable black dispersion was then centrifuged and washed with water three times. A homogenous EGFP-rGO suspension was obtained without aggregation. Finally, the obtained EGFP-rGO sheets were redispersed in water before further use.

### Characterization of GO and EGFP-rGO

GO and EGFP-rGO were characterized according to methods described previously [41]. UV-visible spectra were recorded using a WPA Biowave II spectrophotometer (Biochrom, Cambridge, UK). The particle sizes of the GO and EGFP-rGO dispersions were measured using a Zetasizer Nano ZS90 instrument **(**Malvern Instruments, Worcestershire, UK). X-ray diffraction (XRD) analyses were performed in a Bruker D8 DISCOVER X-ray diffractometer (Bruker AXS GmBH, Karlsruhe, Germany). The X-ray source was 3 kW with a Cu target, and high-resolution XRD patterns were measured using a scintillation counter (*λ* = 1.5406°A). The XRD was run at 40 kV and 40 mA, and samples were recorded at 2θ values between 5° and 80°. The dried powder of GO and EGFP-rGO was diluted with potassium bromide and the Fourier transform infrared spectroscopy (FTIR) (Perkin Elmer Inc., USA) and spectrum GX spectrometry were recorded within the range of 500–4000 cm^_1^. A JSM-6700 F semi-in-lens field emission scanning electron microscope was used to acquire SEM images. The solid samples were transferred to a carbon tape held in an SEM sample holder, and then the analyses were performed at an average working distance of 6 mm. Raman spectra of GO and EGFP-rGO were measured using a WITEC Alpha300 laser with a wavelength of 532 nm. Calibration was initially performed using an internal silicon reference at 500 cm^−1^ and gave a peak position resolution of less than 1 cm^−1^. The spectra were measured from 500 to 4500 cm^−1^. All samples were deposited onto glass slides in powdered form without using any solvent.

### Cell culture and exposure of cells to GO and EGFP-rGO

Human embryonic kidney 293 cells were cultured in DMEM supplemented with 10% FBS and 100 U/mL penicillin-streptomycin in a humidified incubator maintained at 37°C and 5% CO_2_. At approximately 75% confluence, cells were harvested using 0.25% trypsin and subcultured in 75 cm^2^ flasks, 6-well plates, or 96-well plates depending on the intended use. Cells were allowed to attach to the substratum for 24 h prior to treatment. The medium was replaced three times per week, and cells were passaged at subconfluency. Cells were prepared in 100 μL aliquots at a density of 1 × 10^5^/mL and plated in 96-well plates. After the cells were cultured for 24 h, the medium was replaced with medium containing GO or EGFP-rGO at different concentrations (0–100 μg/mL). After incubation for an additional 24 h, cells were analyzed for viability, lactate dehydrogenase (LDH) release, and reactive oxygen species (ROS) generation. Cells not exposed to GO or EGFP-rGO served as the control. Further, morphology of cells treated with GO or EGFP-rGO or untreated was examined using an OLYMPUS IX71 microscope (Japan) using appropriate filter sets.

### Cell-viability assay

The WST-8 assay was performed as described previously [29]. Typically, 1 × 10^4^ cells were seeded in a 96-well plate and cultured in DMEM supplemented with 10% FBS at 37°C under 5% CO_2_. After 24 h, the cells were washed with 100 μL of serum-free DMEM two times and incubated with 100 μL of different concentrations of GO or EGFP-rGO suspensions in serum-free DMEM. After 24 h of exposure, the cells were washed twice with serum-free DMEM, and 15 μL of WST-8 solution was added to each well containing 100 μL of serum-free DMEM. After 1 h of incubation at 37°C under 5% CO_2_, 80 μL of the mixture was transferred to another 96-well plate because residual GO or EGFP-rGO can affect the absorbance values at 450 nm. The absorbance of the mixture solutions was measured at 450 nm using a micro plate reader. Cell-free control experiments were performed to determine whether GO and EGFP-rGO react directly with the WST-8 reagents. Typically, 100 μL of GO or EGFP-rGO suspensions with different concentrations (0–100 μg/mL) were added to a 96-well plate and 10 μL of WST-8 reagent solution was added to each well; the mixture was incubated at 37°C under 5% CO_2_ for 1 h. After incubation, the GO or EGFP-rGO was centrifuged and 100 μL of the supernatant was transferred to another 96-well plate. The optical density was measured at 450 nm.

### Membrane integrity

The cell membrane integrity of human embryonic kidney 293 cells was evaluated by determining the activity of lactate dehydrogenase (LDH) leaking out of the cells according to the manufacturer’s instructions (in vitro toxicology assay kit, TOX7, Sigma, USA) and also as described previously [21]. Briefly, the cells were exposed to various concentrations of GO and EGFP-rGO (0–100 μg/mL) for 24 h, and then 100 μL per well of each cell-free supernatant was transferred in triplicate into wells in a 96-well plate, and 100 μL of the LDH assay reaction mixture was added to each well. After 3 h of incubation under standard conditions, the optical density of the color generated was determined at a wavelength of 490 nm using a micro plate reader.

### Determination of ROS

ROS were estimated according to a method described previously [43]. Intracellular ROS were measured based on the intracellular peroxide-dependent oxidation of 2’,7’-dichlorodihydrofluorescein diacetate (DCFH-DA, Molecular Probes, USA) to form the fluorescent compound 2’,7’-dichlorofluorescein (DCF), as previously described. Cells were seeded onto 24-well plates at a density of 5 × 10^4^ cells per well and cultured for 24 h. After washing twice with PBS, fresh medium containing different concentrations of GO or EGFP-rGO (0–100 μg/mL) was added and the cells were incubated for 24 h. The cells were then supplemented with 20 μM DCFH-DA, and incubation continued for 30 min at 37°C. The cells were rinsed with PBS, 2 mL of PBS was added to each well, and the fluorescence intensity was determined using a spectrofluorometer (Gemini EM) with excitation at 485 nm and emission at 530 nm.

### Statistical analyses

All assays were carried out in triplicate and the experiments were repeated at least three times. The results are presented as means ± SD. All experimental data were compared using the Student’s t test. A p value less than 0.05 was considered statistically significant.
